# Brain-computer interface for robot control with eye artifacts for assistive applications

**DOI:** 10.1038/s41598-023-44645-y

**Published:** 2023-10-16

**Authors:** Kaan Karas, Luca Pozzi, Alessandra Pedrocchi, Francesco Braghin, Loris Roveda

**Affiliations:** 1 Politecnico di Milano, Department of Mechanical Engineering, via La Masa 1, 20156 Milano, Italy; 2 Politecnico di Milano, Department of Electronics, Information and Bioengineering, NearLab, Via Giuseppe Colombo, 40, 20133 Milan, Italy; 3 Istituto Dalle Molle di studi sull’Intelligenza Artificiale (IDSIA), Scuola Universitaria Professionale della Svizzera Italiana (SUPSI), Università della Svizzera italiana (USI), via la Santa 1, 6962 Lugano, Switzerland

**Keywords:** Electrical and electronic engineering, Biomedical engineering

## Abstract

Human-robot interaction is a rapidly developing field and robots have been taking more active roles in our daily lives. Patient care is one of the fields in which robots are becoming more present, especially for people with disabilities. People with neurodegenerative disorders might not consciously or voluntarily produce movements other than those involving the eyes or eyelids. In this context, Brain-Computer Interface (BCI) systems present an alternative way to communicate or interact with the external world. In order to improve the lives of people with disabilities, this paper presents a novel BCI to control an assistive robot with user’s eye artifacts. In this study, eye artifacts that contaminate the electroencephalogram (EEG) signals are considered a valuable source of information thanks to their high signal-to-noise ratio and intentional generation. The proposed methodology detects eye artifacts from EEG signals through characteristic shapes that occur during the events. The lateral movements are distinguished by their ordered peak and valley formation and the opposite phase of the signals measured at F7 and F8 channels. This work, as far as the authors’ knowledge, is the first method that used this behavior to detect lateral eye movements. For the blinks detection, a double-thresholding method is proposed by the authors to catch both weak blinks as well as regular ones, differentiating itself from the other algorithms in the literature that normally use only one threshold. Real-time detected events with their virtual time stamps are fed into a second algorithm, to further distinguish between double and quadruple blinks from single blinks occurrence frequency. After testing the algorithm offline and in realtime, the algorithm is implemented on the device. The created BCI was used to control an assistive robot through a graphical user interface. The validation experiments including 5 participants prove that the developed BCI is able to control the robot.

## Introduction

Robots can be used for a wide range of purposes and with recent advancements in the robotic field, they have become available in many aspects of human life. Robots had a significant impact in many sectors, such as manufacturing operation^1^, teleportation application^2^, intelligent vehicles and air-crafts^3^, entertainment and education^4^, assistive and rehabilitation technology^5^, and robot-assisted surgery^6^. Among these, the assistive and rehabilitation field has the most direct effect on people’s lives. Active prostheses or exoskeletons for assisting different sensorimotor functions such as arm, hand, leg, and ankle are other human-robot interaction applications that are used in assistive technologies^7^. The usage of biological signals has become very common in assistive technologies due to their ability to decode the information of the current state of the human partner. Later, this information can be altered to control devices by the change of measured state. Prosthesis and/or exoskeletons, however, are not an effective solution for people with neurodegenerative disorders, requiring another level of assistance and new ways for human-robot interaction, such as Brain-Computer Interface (BCI).

### BCI state of the art for assistive robotics

BCI is a non-muscular communication channel that enables a person to send commands and messages to an automated system such as a robot or prosthesis, by means of his brain activity^8^. BCI technology has special importance for people with disabilities since people with neurodegenerative disorders may not be able to consciously produce any movements other than those involving the eyes or eyelids^9^. Consequently, BCI offers a good solution for enabling communication with assistive technologies^9–11^. Eye movements and blinks are considered pervasive problems in EEG-based BCI research^12^. However, in some research, eye artifacts have been considered valuable sources of information and exploited for communication and control of machines^13^. Control of a mobile robot with brain-actuated signals is reported in Ref.^14^. Researchers were able to control a mobile robot after a few days of training with EEG-recorded brain signals from standard fronto-centro-pariental positions. Recorded 8 channels were spatially filtered and used in the Welch periodogram algorithm. The resulting signals were used in a statistical classifier to recognize mental tasks. They adopted an asynchronous BCI to avoid waiting for external cues as in synchronous BCI. Evoked potentials were used to control wheelchairs to help people with disabilities, in Ref.^15^. Although it was a slow process, participants were able to choose the location through a graphical user interface. On the monitor, destinations were flashed randomly and when the user focused on a flashed location, the EEG signal presented a peak of around 300 ms. Authors of Ref.^16^ managed to control a robotic arm by a hybrid approach. Users-generated motor imaginary (MI) was used to control the movement of the arm, while P300 potential was used to stop the robotic arm. Two different kinds of evoked potential signals are used in Ref.^17^ for another kind of hybrid BCI system which was developed by using P300 and SSVEP, to control a wheelchair. The problems with the evoked potentials are an external stimulus is required to initiate them and a time loss occurs due to the waiting time between stimulus and detection. Reference^18^ developed a communication interface for people with disabilities. Horizontal and vertical EOG signals were measured using two surface electrodes from either eye and used this information to select the letter from a virtual keyboard based on the thresholding method. Reference^19^ focused on developing a simple motor-controlling application with an EOG sensor for controlling the grasping of a humanoid robot. 2 electrodes, 1 horizontal and 1 vertical, are placed around each eye. They detected blinks by adding a preset minimum threshold and a maximum threshold. In Ref.^9^, authors, contrary to the traditional eye blink detection by using the alpha-blocking in the occipital region measured by EEG, used an EOG sensor and developed a detection algorithm based on thresholding. They collected multiple samples from different people to determine the value. They concluded that the EOG detection’s accuracy is higher than alpha-blocking methods while it requires fewer electrodes to measure. Neural Networks are also used for the detection algorithm in Ref.^20,21^.

In the literature, eye artifact-related papers are generally focused on blink detection, and works using EEG signals are enforcing the participant to not move their eyes. Works focused on eye movement detection are quite rare and they are mostly taking advantage of EOG sensors instead of using EEG. Moreover, eye artifacts have a higher signal-to-noise ratio (SNR) and they are observable in the time domain in contrast to most of the useful EEG signals. The general cons of EOG-based methods are that electrodes placed around the eyes may cause some loss of eyesight and no other information can be gathered compared to an EEG cap.

### Paper contribution

This work proposes a real-time Brain-Computer Interface to control an assistive robot in a human-robot collaborative scenario in order to increase the quality of a patient’s life by enabling interaction with the environment. EEG sensor, the *TMSi SAGA 64+*, will be used to record signals from the prefrontal cortex of the patient. The algorithm will detect the lateral eye movements and blinks that occur voluntarily or forced on the frontal cortex. This information will be used to control a graphical user interface that has multiple functions including controlling the *TIAGo* assistive robot. The contributions of this work to the literature are:

- Creating a unique real-time BCI for *TMSi SAGA 64+* device;
- Developing a novel methodology to detect blinks, by using 2 threshold approach, and left and right eye movements, by exploiting the phase difference and peak and valley pattern, from EEG signals;
- Testing the proposed BCI with online blinks/eyes direction detection to control a real assistive robot (the TIAGo robot).

EEG technology is investigated in this paper instead of EMG, EOG, or contactless eye trackers due to the related advancements in the field in the last years^22^. The aim of this paper, indeed, is to evaluate the possibilities provided by this technology in human-robot interaction applications. In addition, EEG is considered due to its improved comfort and natural human-machine interaction w.r.t. EMG (being placed close to the eye, the solution might be too invasive and uncomfortable) and w.r.t. contactless eye trackers (being affected *e.g.*, by light conditions and head placement).

### Paper layout

The remainder of this work is organized as follows; in Section "Background Information" information related to EEG signals, blink, and eye movement is presented, in Section "Materials and methods" used equipment information about the experimental setup, signal acquisition, processing, threshold detection, and detection algorithm is presented, in Section "Results and discussion" offline and real-time detection results and real-time robot control results are presented, finally, in Section "Conclusions and future developments" conclusion of this work with the possible future works to improve the system are presented.

## Background information

### EEG signals

Brain activity monitoring technologies can be invasive, which requires surgical procedures, or non-invasive. Thanks to its desirable attributes, namely high temporal resolution up to 1 ms, low cost, ease of portability, and non-invasiveness, EEG is the most commonly employed neuroimaging modality^23,24^. EEG signals are typically in the ten to hundred $\mu$ *V* ranges and classified as delta rhythm ($0.1-3.5$ *Hz*), theta rhythm ($4-7.5$ *Hz*), alpha rhythm ($8-13$ *Hz*), beta rhythm ($14-30$ *Hz*), and gamma rhythm ($>30$ *Hz*)^23^. Studies in neuroscience, cognitive science, and psychology fields with EEG devices showed various brain lobes are responsible for specific cognitive activities^25–28^.

**Figure 1:**
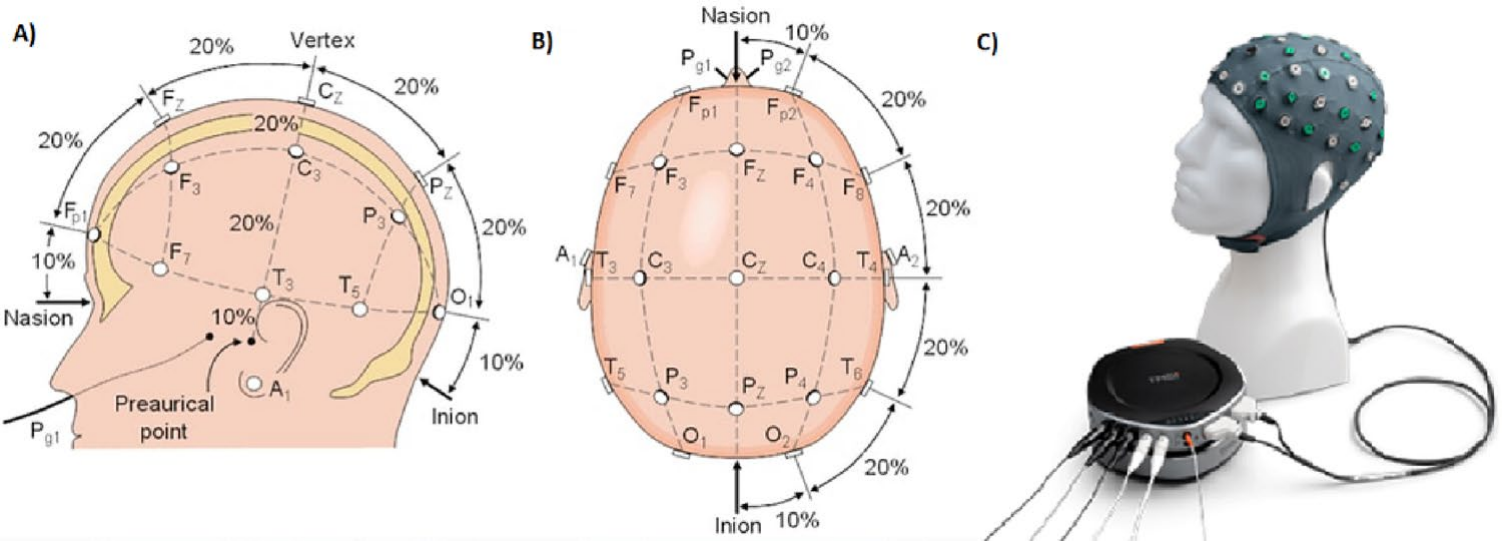
(**A**) the 10/20 System Side View, (**B**) the 10/20 System Top View, (**C**) the SAGA device.

A universally accepted method is used to indicate the locations of electrodes on the scalp, the 10/20 system. The system is reliant on the link between the electrode site and the underlying cerebral cortex region. The frontal, parietal, temporal, and occipital lobes are indicated by the letters F, P, T, and O, respectively and the letter C is utilized only for identification purposes, in the 10/20 system as depicted in Fig. 1 (where the SAGA device and one subject with the SAGA device are also shown). The letter Z (zero) indicates that an electrode is positioned on the midline. Even numbers (2, 4, 6, 8) are used to represent right hemisphere electrode placements, whereas odd numbers represent left hemisphere electrode positions (1, 3, 5, 7)^24^. EEG sensors are small and light, making it convenient to wear them for extended periods of time without being unobtrusive to the users while continuously sensing at home or outside^29,30^.

### Eye blinks and eye movements

**Figure 2:**
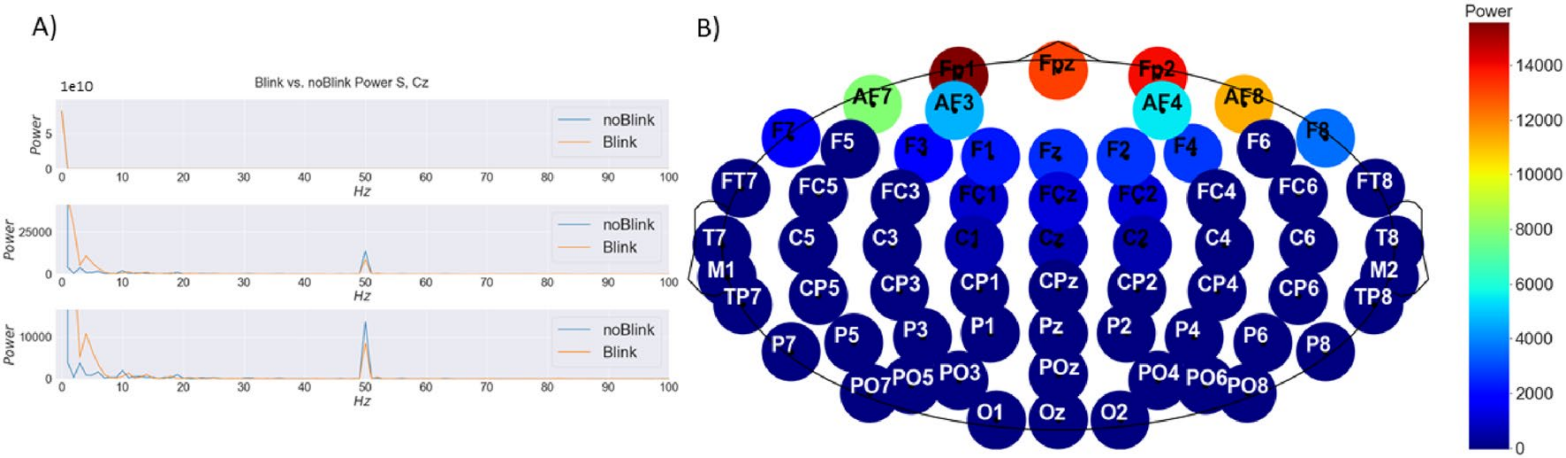
(**A**) Power spectrum of Cz Channel during Blink and no Blink, (**B**) Power topographic of the blink components.

Blinking is the semi-automatic closure of the eyelids and it protects the eyes from potentially harmful stimuli such as bright lights and foreign substances, like dust while it does not affect the continuity of the subjective impression of images. Various eye-related motions, such as lateral movement to the left, right, up or down, blinks, and so on, give rise to characteristic features on the signals collected^31^. The eye blink signal acquired by EEG electrodes, due to the electrodes’ different positioning, is different from the same event recorded by electrooculography (EOG)^31^. Eye blink and eye movement power spectrum are investigated at various channels and it is found that when a blink or eye movement occurs a change of the signal power can be observed in delta ($0.1-3.5$ *Hz*), theta ($4-7.5$ *Hz*) and alpha ($8-13$ *Hz*) bands^21,32–34^. Alpha rhythm blocking, which is the increase of brain signal amplitudes in alpha bands due to the closure of the eyes in a wakeful condition, is used to detect blinks at various channels and eye closing/opening procedure has a widespread effect overall EEG electrodes^9,35^. In^36^, authors demonstrate the effect of a blink in C3 channel in the averaged power spectrum. Power corresponds at frequencies between 0 to 30*Hz* is higher for blink compared to no blink and this phenomenon can be observed in all channels even without averaging multiple times of the same event. An example of this behavior is demonstrated in Fig. 2A) for Cz Channel. In another work authors of^37^ demonstrated the blink power topographic distribution on the scalp. The power decreases while moving away from the source, eyes. The same phenomenon is also demonstrated in Fig. 2B) with fewer channels.

**Figure 3:**
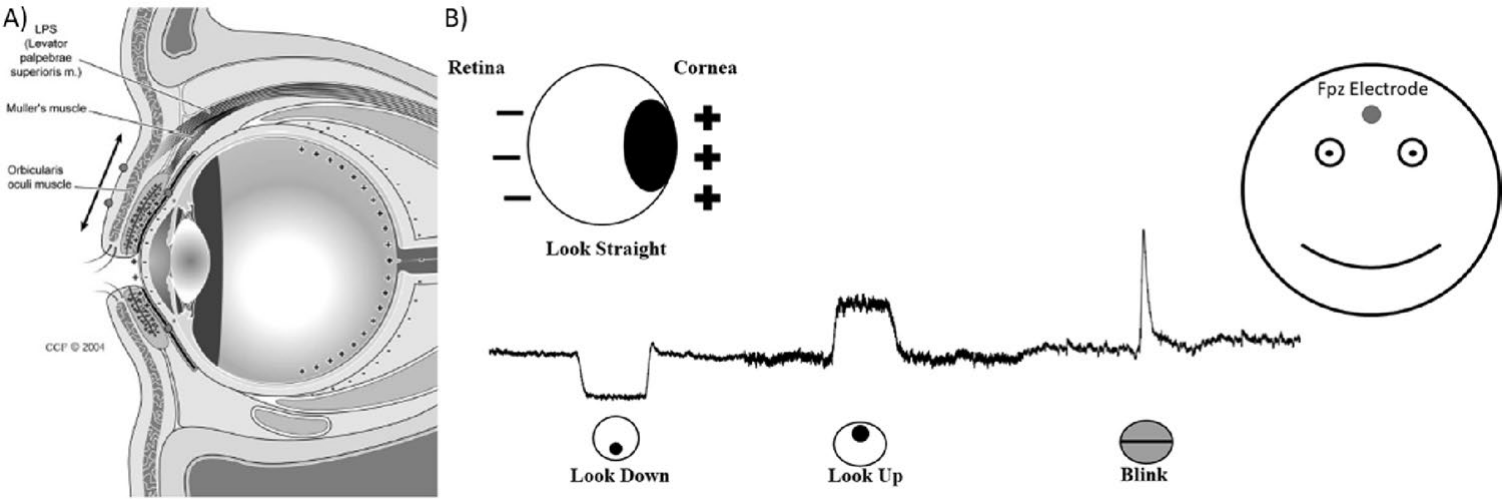
(**A**) Anatomy of eye and eyelid, shown in a parasagittal section^44^ (**B**) Polarity of Retina and Cornea and its effect on Fpz Channel on EEG Signal.

In addition to the change in the power of the signal, a spike on the signal can also be observable when there is a blink and in the case of movements, multiple spikes are observed. Corneo-retinal potential (CRP) refers to the constant potential difference that exists between the retina and the foveal sclera of the eye. This facilitates the generation of a large-amplitude current field with the movement of eyeballs in every direction. With the frontal electrodes that are placed in accordance with the 10-20 System, it is possible to detect those current fields, as demonstrated in Fig. 3. When the eyeball rotates upwards, the positive pole (cornea) becomes closer to the frontal channels and produces a positive deflection. On the other hand, a downward rotation causes a negative peak in those channels. This is identical to what happens when a blink happens. When the eyelid shuts, the cornea moves closer to Fp1, resulting in a positive deflection. However, as the eyelid opens, the cornea rotates away from electrodes, resulting in a negative deflection^32,34,38,39^. Another form of the electric signal is produced by eye movements. The cornea of the eye is positively charged relative to the retina, resulting in a continuous retino-corneal charge of between 0.4 and 1.0*mV* in both eyes, which approximates a dipole. The direction of this dipole in three-dimensional space shifts when the retino-corneal axis rotates during eye movements, resulting in variations in electric potential. Signals caused by eye movement travel mostly through the shunt conduit given by the eye sockets. These signals attenuate more slowly than blink signals^40^. Opposite polarity is induced during lateral eye movements in the left and right hemispheres, resulting in the phase difference^18,32^. Duration of the eye blink can vary between 200 to 400*ms* and its electrical magnitude is more than 10 times that of cortical signals but it rapidly decreases with the distance from the eyes^40–42^. The electrical magnitude of the blinks has some fluctuations within the same individual and even higher variation amongst different participants^43^. Eye blinks can be classified into three types: reflexive, spontaneous, and voluntary^18,39,43^. The reflexive eye blink is the simplest response and does not engage cerebral regions^21,43^. The spontaneous blink (also referred to as involuntary or natural eye blink) happens ten to twenty times per minute without external stimulation and serves to clean, lubricate, and oxygenate the cornea^31,43^. The voluntary blink is caused by intentional eye closing and it produces clearer signals with larger amplitude than that obtained from other types of blinks and lateral eye movement^18,43^. Moreover, it involves multiple areas of the cerebral cortex^21,43^.

## Materials and methods

### Materials

In this work, a 64-bit Windows laptop is used to record EEG data for offline analyses. The device has 16 GB of installed RAM, 15.8 GB is available, and has an Intel(R) Core(TM) i7-6700HQ with a 2.6 GHz base and 3.1 GHz turbo CPU. The processor has 8 cores for multiprocessing. *TMSi SAGA 64+* device is used for EEG signal acquisition, sampling frequency was chosen as 500 *Hz*. A gel-type EEG cap was used and in all experiments, impedance values were kept under 5 k$\Omega$. The ground electrode was placed at the bone back of the right ear and the reference electrode at the bone back of the left ear.

### Participants

All the experiments that involved human subjects were approved by the ethical committee of Politecnico di Milano (Opinion n. 13/2021), and participants were asked to sign a written informed consent.

All the experiments were carried out in accordance with relevant guidelines and regulations.

5 subjects (3 males, 2 females, age $27\pm 3$), participated in experiments controlling a robot platform (Section 4.4) with a given consent. During all sessions, the subjects were asked to sit comfortably in front of the monitor. The eye side is aligned with the center of the screen so that the idle rhythm is not affected. At the beginning of each trial, the desired eye artifacts are told to the participant.

### Signal acquisition

In order to decide which channels to use, channels corresponding to the frontal, temporal, parietal, and occipital cortexes are examined. After a thorough investigation of raw and filtered ( band pass filtered between $0.5 - 100$ *Hz*) signals, FP1, F7, and F8 channels were decided to be used in the detection algorithm. FP1 channel demonstrated the highest relative peaks compared to other channels and it is used in the blink detection part, while F7 and F8 channels showed the highest relative change during the lateral eye movement and consistent phase difference compared to other coupled channels on opposite hemispheres and they are used in the detection of lateral movements.

### Signal pre-processing

**Figure 4:**
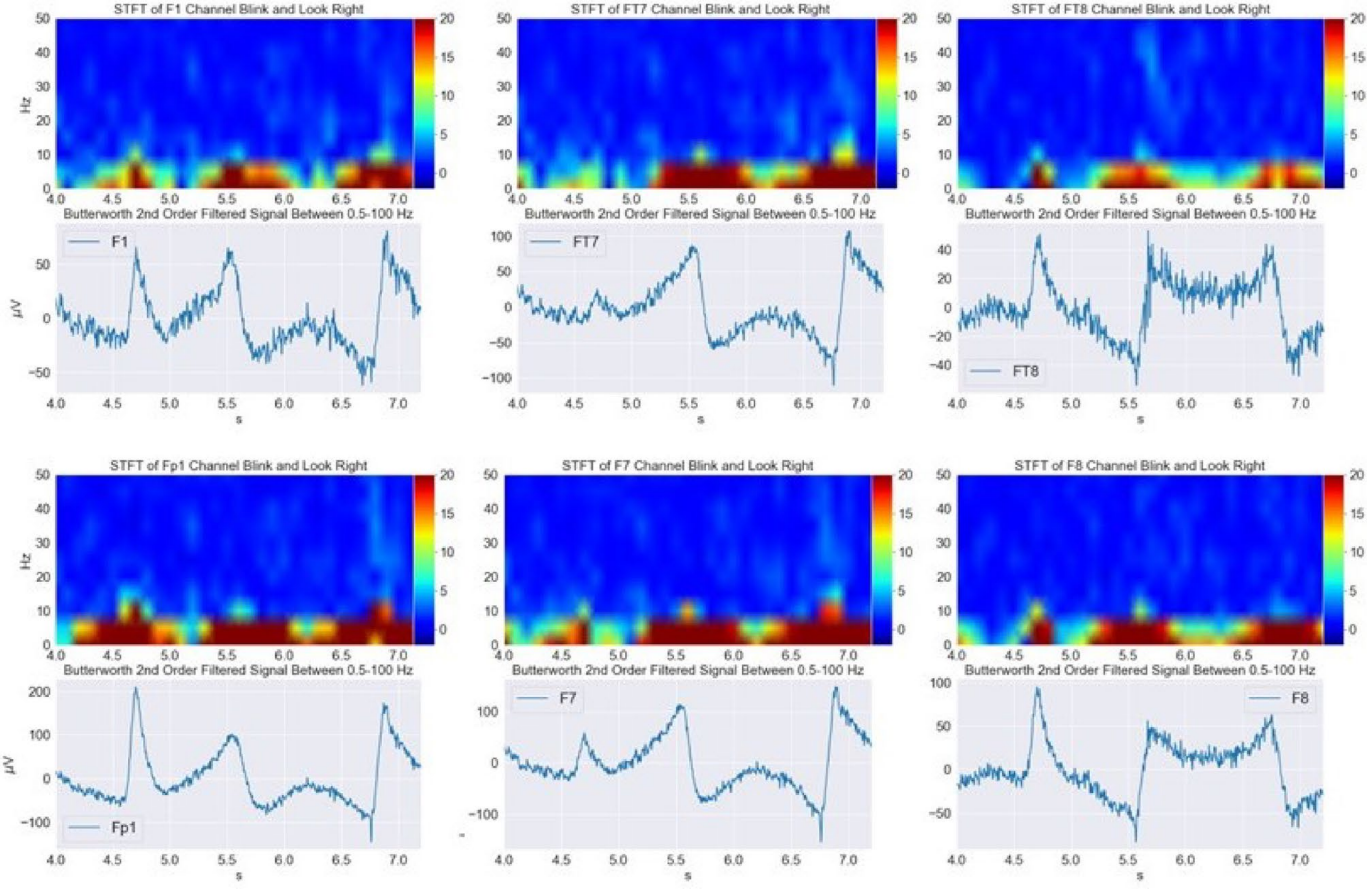
STFT of F1, FT7, FT8, Fp1, F7, F8 channels while blink and look right on 22/07/2022.

In the literature, as has been mentioned before, eye artifacts elicit power increases in specific frequency bands. There are different suggestions for the frequency band between $0-13$ *Hz*^21,33,38^. In order to find a relevant frequency band and affirm the previous findings, time-frequency analyses of the channels were conducted. Short-time Fourier transform (STFT) is used to determine the sinusoidal phase and frequency content of local sections of a signal as it changes over time. The signal is segmented in windows of 100 data points with an overlap of 50 data points. Hanning windows have been applied. Before calculating the STFT, the signal is filtered to get rid of the high-frequency components, higher than 100 *Hz*, and low-frequency components, lower than 0.5 *Hz* which are in line with the mentioned frequency bands of the EEG signal. Frontal channels showed more clear results compared to others. The rise in the amplitudes is exactly in line with the events, demonstrated in Fig. 4. By looking at electrodes in the temporal cortex, a rise of power between $0.5-15$ *Hz* is observed and in the frontal cortex, it has a frequency range of up to 30 *Hz*.

**Figure 5:**
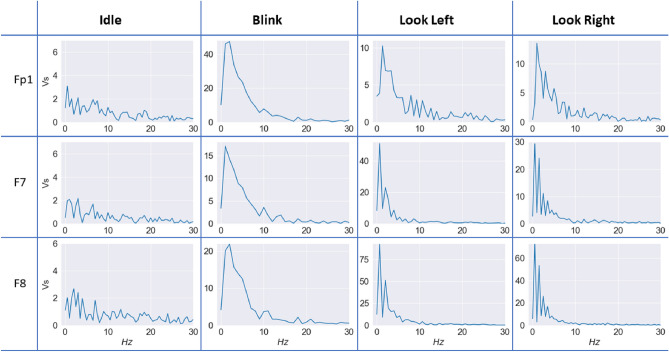
FFT of Idle, Blink, Look Left and Look Right at Fp1, F7, F8 Channels on 28/06/2022.

Fourier transform (FT) of the idle, blink, look left, and look right events are calculated to further examine the components of the signal between $0.5-30$ *Hz*. After visualization of the FT plots, Fig. 5, it is clear that the dominant frequency bands are indeed between 0.5 and 15 *Hz*. Since the principal frequency bands are prior to 15 *Hz*, a second-order Butterworth filter between $1-13$ *Hz* is chosen for filtering the signal.

#### Remark 1

Blink, look left, and look right signals have been considered in this paper. Indeed, look up and look down are not studied. These signals can be included in future work to provide more capabilities to the proposed approach.

### Threshold determination

**Figure 6:**
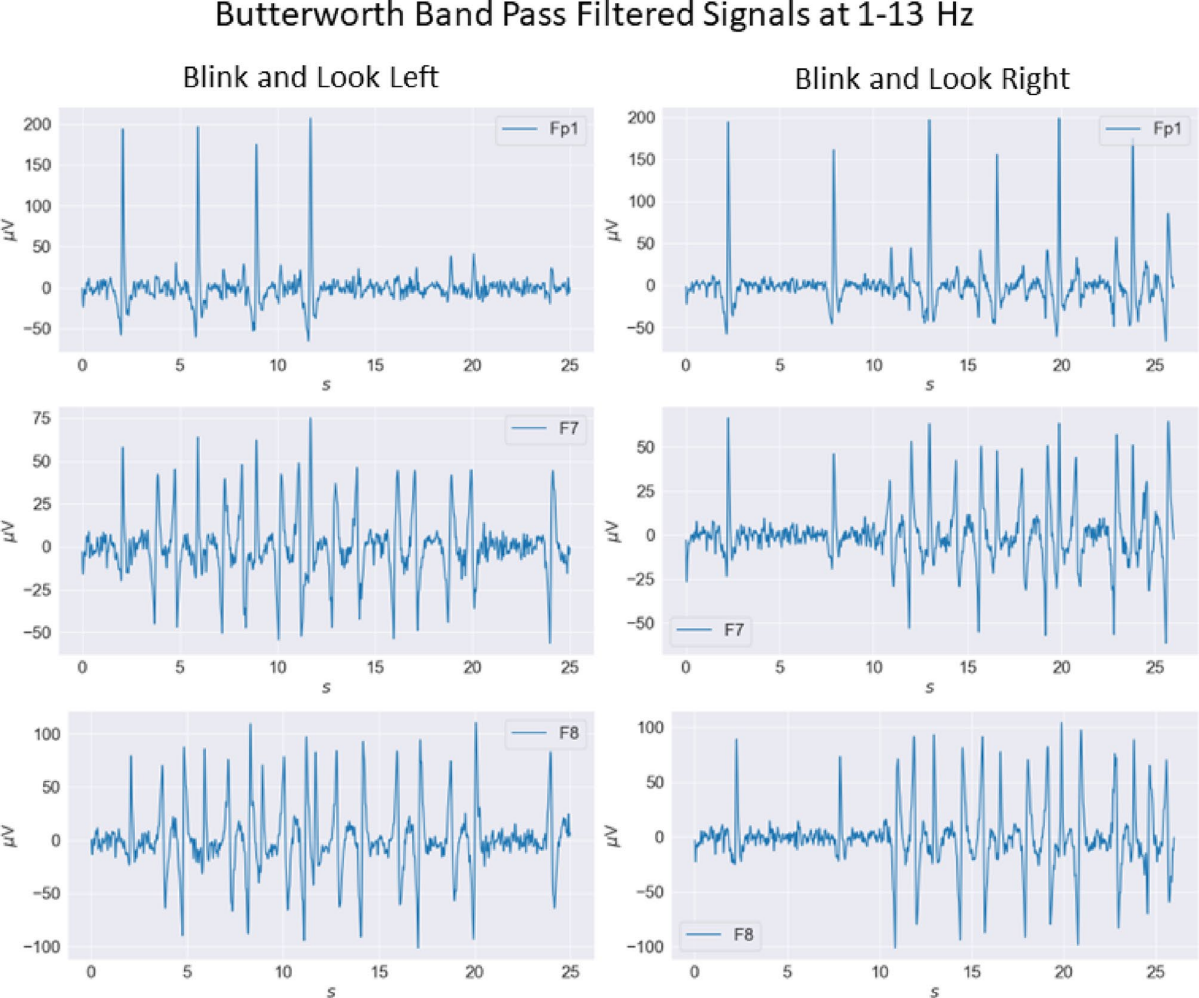
Butterworth Band Pass Filtered Signals at 1–13 Hz of Channels Fp1, F7, and F8 on 28/06/22.

Most of the threshold-based approaches suffered from the variability of peaks between subjects and also within subjects on different dates^45^. By considering these, threshold determination was done by considering all experiments at one date, and then to reduce the variability within a subject, different dated experiments were used to optimize the final values. Two values were chosen for blink for the Fp1 channel and 4 values for the look left for F7 and F8 channels and 4 values were chosen for the look right for F7 and F8 channels. The general behaviors of the signals on the events are demonstrated in Fig. 6.

The mean and standard deviation values for each trial are calculated separately as demonstrated in Eq. (1) and distribution analysis was conducted to investigate the quartiles and outliers of mean values for all peaks and valleys. Considering the mean and standard deviation the threshold value should be between $167-22\alpha$ $\mu V$ and $167+22\alpha$ $\mu V$ for the Fp1 channel to detect blinks. For significant peaks, considering the relation given above, $\alpha$ was chosen to be 0.5 so that the first threshold became 156 $\mu V$. The value also corresponds to the first percentile of the overall distribution. For the second threshold, $\alpha$ was chosen to be 4 based on the visual examination of all data sets, and the second threshold was determined as 80 $\mu V$, which is lower than all the blink peak values during the experiments.

1 $$\begin{aligned} THR&= M - \alpha *SD, \end{aligned}$$

2 $$\begin{aligned} M&= \frac{\sum ^N_{n=1}{y(n)}}{N}, \end{aligned}$$

3 $$\begin{aligned} SD&= \frac{\sqrt{\sum ^N_{n=1}{(y(n)-M)}}}{N}. \end{aligned}$$

**Figure 7:**
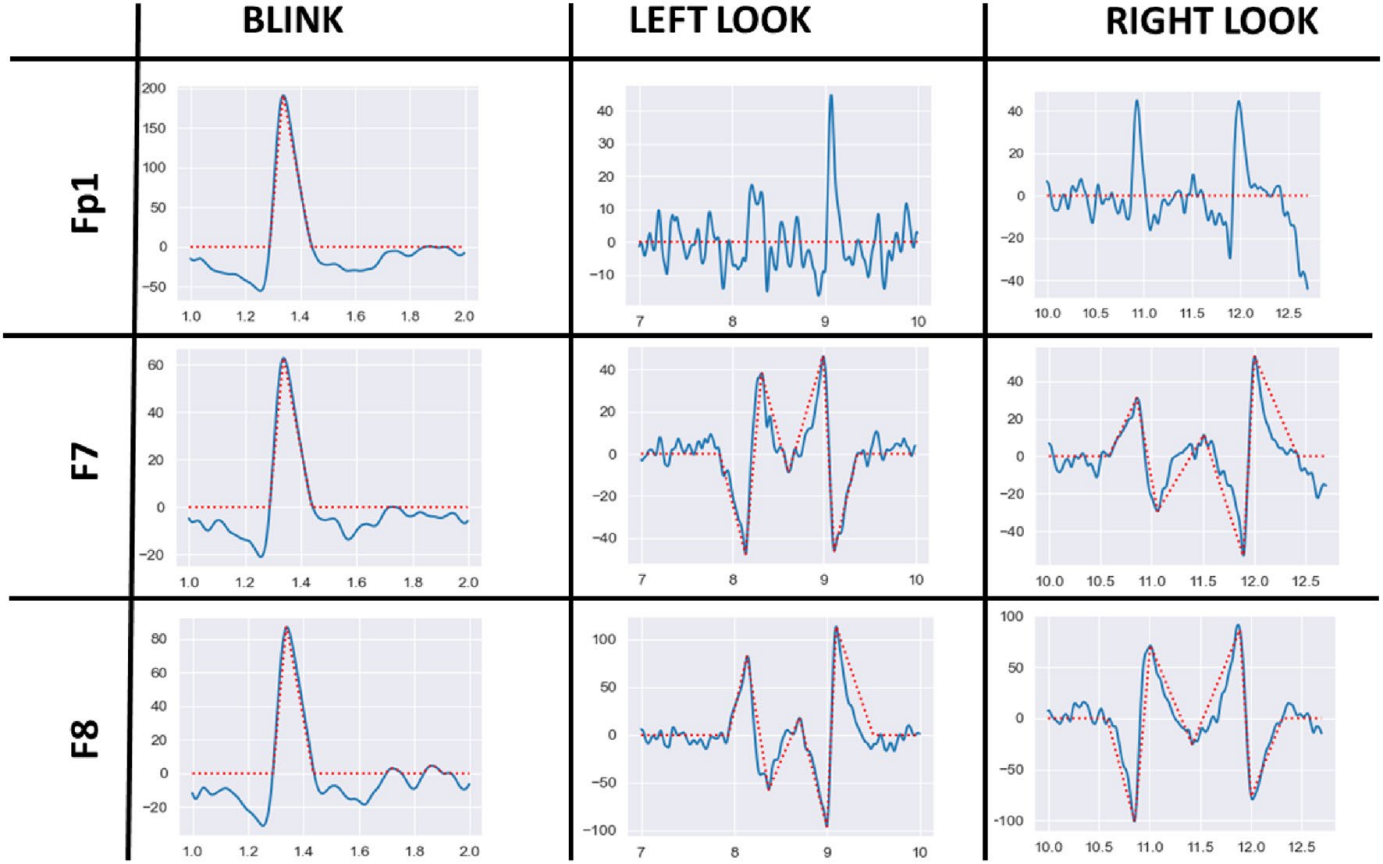
Effects of Eye Artifacts on Fp1, F7 and F8 Channels.

A similar procedure was followed to find threshold values for F7 and F8, only this time both negative and positive peaks have to be found (all the thresholds are shown in Table 1). As it is demonstrated in Fig. 7, left and right looks have an opposite behavior which is composed of multiple peaks and valleys. The peak at one channel and the valley in the other were observed almost at the same time. The mean and standard variation of each channel for each trial is calculated separately. Then the distribution of the peaks and valleys is examined to determine thresholds.

**Table 1:** The threshold values set for each channel, and the corresponding event.

Channel	Event	Threshold [$\mu V$]	
		Upper	Lower
Fp1	Blinks	156	80
F7	Look left	20	− 20
	Look right	20	− 20
F8	Look left	30	− 20
	Look right	20	− 30

### Algorithm

The algorithm can distinguish single blinks, look left and look right events. Furthermore, it also catches the relative time, the first data point received corresponds to 0 in virtual time. Filtered data is divided into windows of size 100 and fed into the algorithm.
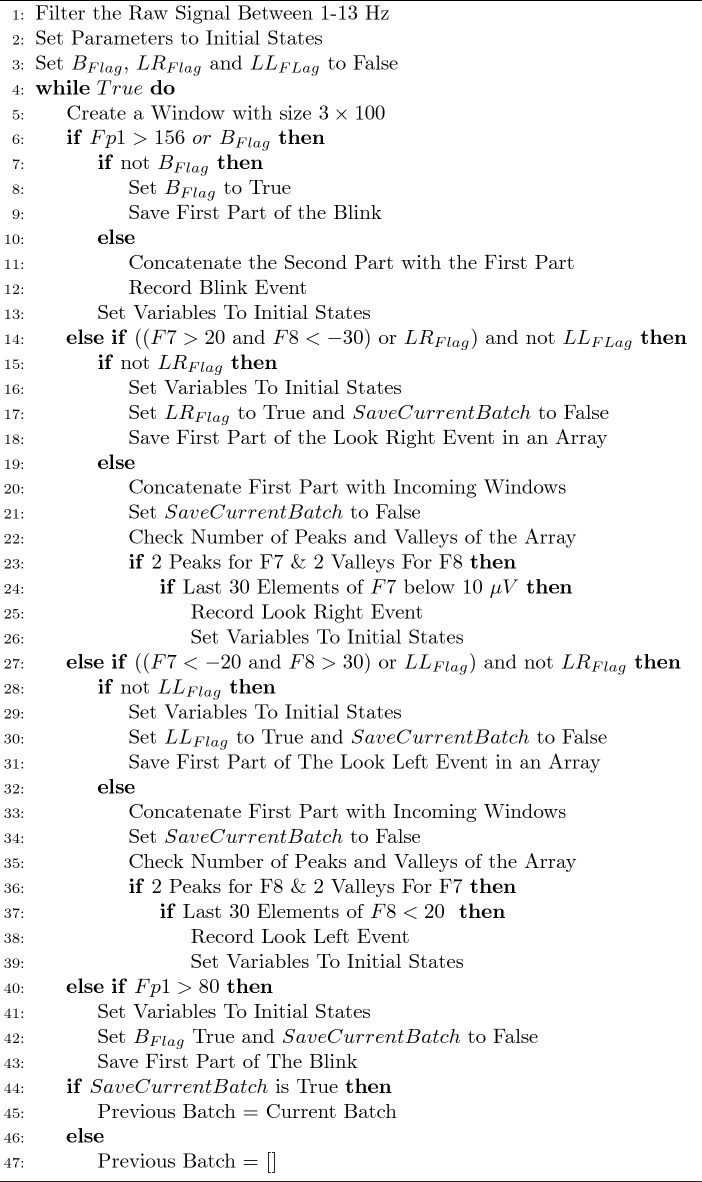


First, the algorithm checks whether the Fp1 channel has a value higher than 156 $\mu V$ and if there is, it raises a *Blink Flag*. If there is a previous window, that is not used in any event, it is added before the new event. Since *Blink Flag* is raised, the next batch also enters to blink case. This allows passing the threshold obligation so that the remaining, downward decrease from the peak of the signal, can be caught. The new window is concatenated with the previous one in the previous variable. Since a blink can occur between 200 ms to 400 ms, this enables the algorithm to catch long blinks. If a blink event is caught, then the last batch of data is not kept for the next run.

In the second case, the algorithm checks for F7 and F8 channels. If there is a value higher than 20 $\mu V$ for F7 and lower than $-30$ $\mu V$ for F8 channels, the algorithm initiates the detection of the look right event by raising *Look Right Flag*. Unless a blink occurs, all new batches enter to look right case. The previous batch, if it is not used, merged with the current batch. The main idea of the algorithm is to increase the size of the window while checking the peaks and valleys of both F7 and F8 channels. Recorded window loops in the same condition with new batches concatenated in each loop until it acquires the desired shape for corresponding channels. After it succeeds, it checks whether the tail of the signal diminished within the idle state by examining the amplitudes of the last elements of the window. Then the algorithm exits the case and all flags turned to the initial state. Look left case is similar to the right case, the only difference is the order of the peaks on corresponding channels. Finally, if a current sample does not enter any of those statements, an additional conditional statement checks whether a weak blink has occurred. If the statement detects a value higher than 80 $\mu V$ inside the Fp1 stream of the data, it raises the *Blink Flag*. Since the *Blink Flag* is raised, the next batch will enter the first condition and a blink event will be caught. Lastly, if the batch does not satisfy any of the conditions, it is recorded to become the previous window to be used with the next batch. If the next batch also does not satisfy any conditions, then the old one is deleted and the current one takes its place.

In the next chapter, offline and real-time test results will be demonstrated. Furthermore, the BCI application, controlling *TIAGo*, through a graphical user interface is also explained in the "Results and discussion".

### Data flow and processing schema

**Figure 8:**
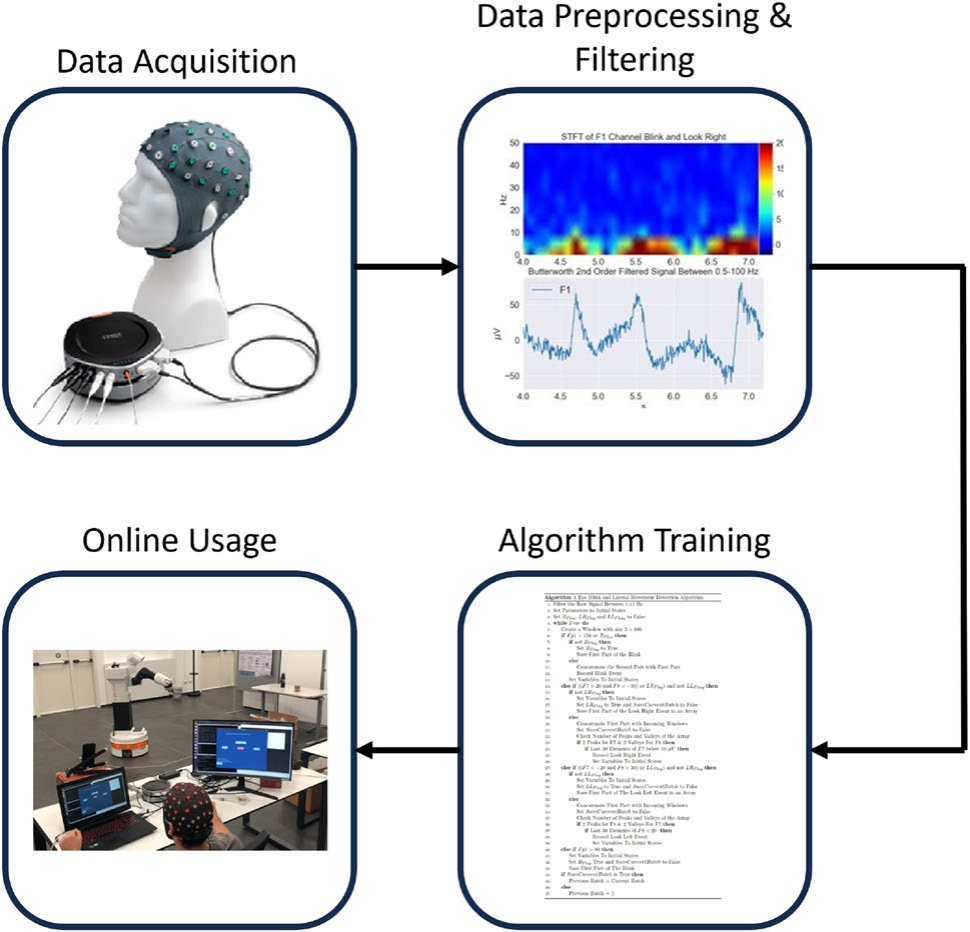
The data flow and processing schema is shown. The data are acquired from the SAGA device. Then, the data are preprocessed and filtered. The training algorithm is fed with the processed data. Finally, the trained algorithm is used online in the human-robot interaction application.

Figure 8 shows the data flow and processing schema, including the proposed algorithm for eye motion classification.

## Results and discussion

The performance of the developed system was evaluated by using offline data which was gathered through a series of experimental tests performed with the same subject. After obtaining satisfactory results with offline data, real-time tests were conducted, with the same participant, and the performance of the algorithm was evaluated. After observing satisfactory results both in offline and real-time tests, the algorithm is implemented to communicate with *TIAGo* through a graphical user interface to achieve successful robot control. 5 participants tested the developed algorithm, controlling the TIAGo robot with the proposed BCI approach.

### Offline results of the algorithm

**Table 2:** Offline detection accuracy of the algorithm.

Experiment	Blink		Look left		Look right	
	Accuracy	$\%$	Accuracy	$\%$	Accuracy	$\%$
15/06/22	206/299	90.0	11/13	84.6	14/18	77.8
16/06/22	164/208	78.8	25/27	92.6	22/28	78.6
28/06/22	88/94	93.6	47/49	95.9	49/50	98
29/06/22	102/102	100	58/59	98.3	68/71	95.8
20/07/22	111/137	81.0	72/74	97.3	49/62	79
22/07/22	137/141	97.2	68/68	100	54/67	80.6
Overall	808/911	88.7	281/290	96.9	256/296	86.5

6 different dated sets of data were examined to determine offline performance. In Table 2, the results of the offline system are demonstrated. EEG signal variability in the same subject can be observed from the results. The setup of the system, electrode placement of the scalp, and impedance values of the electrodes have a high influence on the results as well as the natural variation in biological conditions of the patient, such as skin conductance, and eye moisture. Nevertheless, the overall performance of the offline system was quite satisfactory. The start and the end of the events can become decisive if two events occur close to each other. In some of the cases, in which two eye motions or an eye motion and a weak blink occurred without the initial one settling down, the first event was recognized but the first recorded event also contains the second one inside. Due to this, misclassification or misses were observed. The confusion matrix is demonstrated in Table 3. From the table, it can be concluded that some cases of looking right and blink events are misclassified as looking left. However, these results do not mean that one event is misclassified as another event but multiple events were forming a single misclassification. Overall the 70 blinks, 5 look right, and 9 look right events were not recognized. After observing satisfactory results with offline setup, the algorithm was implemented into the *TMSi SAGA 64+* data acquisition library.

**Table 3:** Confusion matrix of overall offline detection tests.

	Actual blink	Actual look left	Actual look right
Predicted blink	808	0	7
Predicted look left	29	281	24
Predicted look right	4	4	256

#### Remark 2

As highlighted in this section, 6 datasets from a single subject and different days have been used to train the algorithm. This makes it possible to account for uncertainties and variability of user behavior, achieving more robust training. In the same way, data belonging to multiple users can be included in the training, providing robust behavior across different subjects. If the algorithm’s performance has to be improved, transfer learning or meta-learning approaches might be considered. As a matter of example, fine-tuning for a specific user can be achieved on the basis of Ref.^46^, where a Recurrent Neural Network (RNN) is cascade-connected with a Fully Connected layer retrained for each specific subject in a human-robot collaborative application.

### Real time results of the algorithm

In real-time tests, the participant was allowed to perform eye artifacts by his choices. The experimental data were also saved for offline results to compare accuracy. In order to use the algorithm in real-time, *Python* library which is provided by *TMSi* company for data acquisition has to be modified. As far as the authors’ knowledge, there was not any available open-source or provided real-time processing software for this device. The provided library has the function for semi-real-time filtering of the signal for visualization purposes. The function provided was exploited and by introducing new functions, including a detection algorithm, real-time data processing and detection of the eye artifacts was made possible with the *TMSi SAGA 64+* device.

Overall in the real-time tests, the subject performed 316 blinks, 82 left look, and 83 right look movements. Afterward, the recorded experimental data sets were fed into the offline algorithm to compare the results. In Table 4, both results are demonstrated. Offline and real-time performance of the same data showed quite different results, with the real-time application having lower accuracy. One of the main reasons for this difference can be the difference in filtering between both cases. In real-time operation, random-sized signals are windowed compared to offline where the whole signal is filtered at once. Although real-time accuracy was lower than expected, it was enough to control an assistive robot through a GUI since moving between buttons can be done with both lateral sides and the lacking one can be suspended by the other one. As far as the blink detection, its accuracy does not vary much between real-time and offline algorithms, since it has more evident peak amplitudes.

**Table 4:** Real time vs. offline detection accuracy.

Experiment	Blink		Look left		Look right	
	Accuracy	$\%$	Accuracy	$\%$	Accuracy	$\%$
09/09/22$^{1}$	25/25	100	–	–	–	–
09/09/22$^{2}$	25/25	100	–	–	–	–
14/09/22$^{1}$	188/189	99.5	43/59	72.9	27/58	46.6
14/09/22$^{2}$	189/189	100	56/59	94.9	58/58	100
23/09/22$^{1}$	96/102	94.1	6/23	26.1	16/28	57.1
23/09/22$^{2}$	102/102	100	17/23	73.9	23/28	82.1
Overall$^{1}$	309/316	97.9	49/82	59.8	43/86	50.0
Overall$^{2}$	316/316	100.0	73/82	89.0	81/86	94.2

$^{1}$real time detection.

$$^{2}$$offline detection.

### BCI experiment description

**Figure 9:**
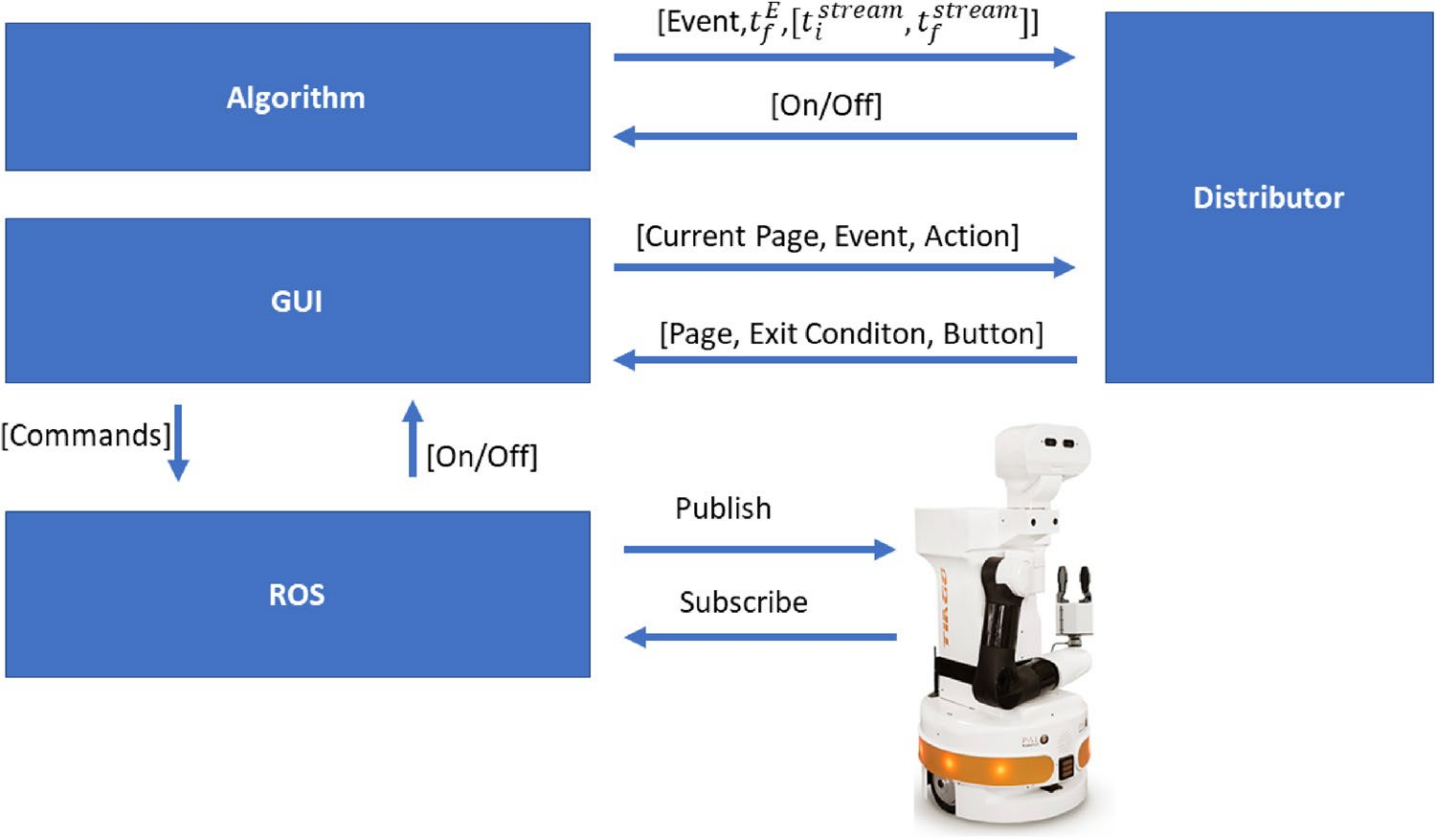
Communication flow between subsystems.

For the robotic unit, *TIAGo* by *PAL ROBOTICS* was chosen. Robot communication is possible through *ROS*, Robotic Operating System, *Melodic* framework. In order to have full access to all offered features, *Ubuntu 18.04* operating system was used as a second operating system on the same computer. In order to create a successful BCI communication, some subsystems had to be created, namely an event-detection *algorithm* (as described in subsection 3.6), a *distributor* to further differentiate multiple blinks (single, double, or quadruple blinks) based on the frequency of occurrences, a graphical user interface (*GUI*) through which the user can select the desired task, and a *ROS* distributor, enabling communication between the described pipeline and the ROS network that controls the robot. In Fig. 9, the data flow within the systems is demonstrated. By considering the initial setup of the GUI, the distributor sent the queue to switch between buttons or change pages by incoming events from the device. Double blink is used to press buttons for changing the page, picking the object, or in the robot base control page, going forward. On the other hand, lateral movements are used to move between buttons. Continuous rotation is adopted for changing the highlighted button. Finally, 4 consecutive blinks are used to go back to the previous page. In order to detect double and quadruple blinks, the virtual time of the detected event and the latest stream are used. The algorithm sets the time between blinks as 0.5*s* second, and it waits for 1.5*s* second from the first occurrence of a blink to make the final judgment whether it is a single, double, or triple blink. If a quadruple blink is initiated within the time frame then it directly sends the appropriate command, *“return to the previous page”*, to the GUI.

GUI welcomes the user with 2 options; controlling the mobile base of the robot and the other is choosing a predefined task:

- On the mobile base control page, the robot can either go forward for 1*m* or rotate left or right by $15^{\circ }$.
- From the predefined tasks page,
  - A caregiver can be informed through email;
  - The robot can perform a dance which also resets its arm, head, and torso position and aligns it with the initial direction;
  - The robot can go to a table at a predefined location;
  - The robot can scan the plane in front of it to distinguish the objects on it. The currently selected object is highlighted and the user can change the selection by looking left or right. At this stage, the pick task is activated. After pressing the pick button by double clicking, the robot calculates its arm and torso joint values and picks the object. After the robot grips the object, it goes back to a safer spot since the robot arm configuration is different from the initial state. Then a participant can control the robot to any position from the base motion page.

### BCI validation experiment

**Figure 10:**
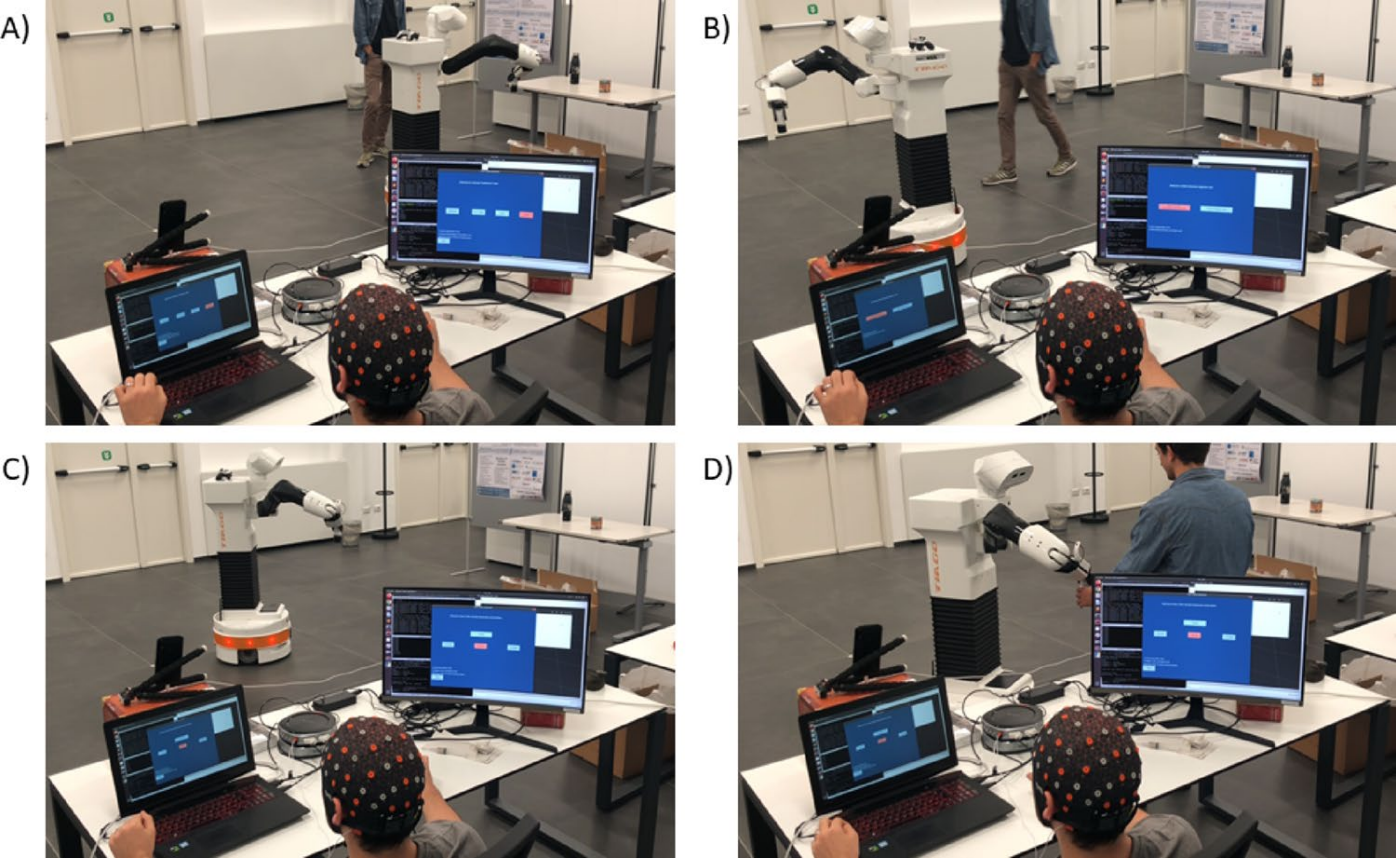
(**A**) *TIAGo* moving back with the object, (**B**) *TIAGo* reached a safe position, (**C**) Participant moving the base of *TIAGo*, (**D**) Colleague taking the object from the *TIAGo*.

**Figure 11:**
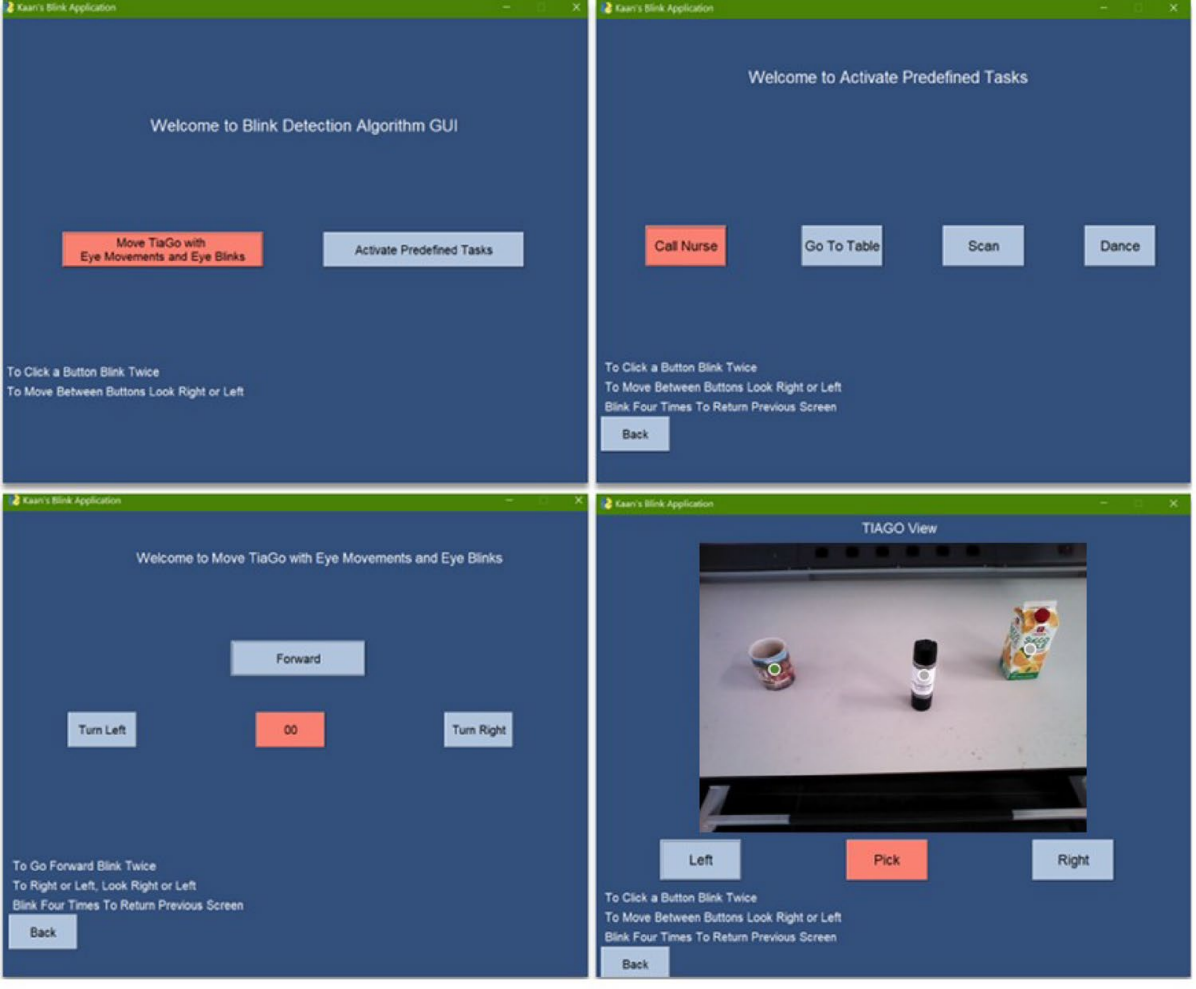
Implemented GUI for BCI.

In this section, the performance of the BCI robot control with eye artifacts is presented. The participants were able to control the base movements and select a predefined task for the robot to perform by using their eye artifacts. The subjects tested all functions of the robot to validate the usability of the whole system. Figure 10 demonstrates some frames from the video of the validation experiment of the BCI system. Figure 11 shows the implemented GUI for BCI.

The validation tests prove that indeed the developed novel thresholding-based pattern recognition algorithm for detecting eye artifacts from EEG signals was able to control a robot through eye artifacts. In the following Tables 5, 6, 7, 8, 9, the confusion matrix for detection of blinks, look left, and look right in the real-time experiment is presented for all the subjects.

**Table 5:** Confusion Matrix of Overall Real-Time Experiments - Subject #1.

	Actual blink	Actual look left	Actual look right
Predicted blink	901	11	42
Predicted look left	6	41	7
Predicted look right	44	112	201

**Table 6:** Confusion Matrix of Overall Real-Time Experiments - Subject #2.

	Actual blink	Actual look left	Actual look right
Predicted blink	788	23	47
Predicted look left	9	34	8
Predicted look right	33	56	101

**Table 7:** Confusion Matrix of Overall Real-Time Experiments - Subject #3.

	Actual blink	Actual look left	Actual look right
Predicted blink	921	44	71
Predicted look left	10	55	12
Predicted look right	65	113	199

**Table 8:** Confusion Matrix of Overall Real-Time Experiments - Subject #4.

	Actual blink	Actual look left	Actual look right
Predicted blink	895	37	76
Predicted look left	9	39	10
Predicted look right	79	117	192

**Table 9:** Confusion Matrix of Overall Real-Time Experiments - Subject #5.

	Actual blink	Actual look left	Actual look right
Predicted blink	905	39	81
Predicted look left	13	55	11
Predicted look right	87	113	179

The eye artifact detection algorithm showed poor results in detecting left looks and a lot of misclassification of look left as look right has been observed. However, the subjects were able to perform all tasks.

### Discussion

The algorithm is designed and presented as both an offline and a real-time algorithm. Real-time identification of eye artifacts broadens the applicability of such a method in the domains of BCI-based communication, control, neurogaming, as well as real-time EEG data processing. By design, the algorithm records the eye artifacts and occurrence time of the events which enables the researcher to later examination of the events. Although the offline results are accurate, during the online process the overall accuracy drops a lot. The reason for that can be the filtering operation in the real-time case is done to small and different-sized matrices and then they combined to form a sample with 100 data points. Later, it is used in the algorithm. On the other hand, in the offline case, the whole recorded data is filtered and then it is split into 100 samples and sent to the algorithm one batch at a time. Real-time filtering can be done after collecting the 100 data points so the accuracy may increase. Another issue with the algorithm is that after each lateral movement, a small amount of time had to be waited to increase the accuracy. However, this does not represent an issue for the application at hand as, for the robot base movements, the user has to wait for the robot to complete each motion before giving a new one to establish aware control of the robot. For other kinds of applications like neurogaming, this might create some problems due to the reduction of reaction time. Nevertheless, this real-time BCI algorithm to control a robot with eye artifacts can improve the life of a disabled person based on the results exhibited in this work.

## Conclusions and future developments

The thesis work, presented in this paper, proposes a BCI robot control with eye artifacts for people with disabilities to improve their lives by giving them a method to interact with the environment through an assistive robot. The developed algorithm detects eye artifacts through characteristic shapes of the EEG signals. The lateral movements are distinguished by their ordered peak and valley formation and the opposite behavior of F7 and F8 channels. This work, as far as the authors’ knowledge, is the first method that used this behavior to detect lateral eye movements. For the detection of blinks, a thresholding method is proposed but as different from the works in literature, two thresholds are determined to catch the weak blinks as well as regular ones. The real-time detected events with their virtual time stamps are fed into a second algorithm, distributor, to further differentiate the consecutive blinks based on the frequency of occurrences to determine whether users perform single, double, or quadruple blink.

The proposed algorithm was tested offline to assess the performance of the blink, look left, and look right detection. Variability of the EEG signal of the same participant on different dates causes the performance to vary. Nevertheless, the proposed algorithm has been proven to detect eye artifacts. The worst observed accuracy results with the record data sets are $77.778 \%$ for a look right, $84.615 \%$ for a look left, and $78.846 \%$ for a blink. The variation of the EEG signal between different subjects has to be further investigated to demonstrate the generality of the algorithm. After observing the offline results, the algorithm is embedded inside the acquisition algorithm, which is one of the first real-time EEG signal processing algorithms with this device, to check its performance, and a BCI is developed to control an assistive robot. Finally, with the developed BCI, the robot performs each task successfully.

The algorithm is designed and presented both offline and online. By design, the algorithm records the eye artifacts and occurrence time of the events which enables the researcher to later examination of the events. One of the directions to extend this work is to feed the recorded events into a neural network, such as a supervised clustering algorithm, which is trained with a blink, look left and look right events so that even if a miss classification occurs, the second layer can catch or correct the output. It is a challenging task to add a second layer into the general process, since the event occurrence and output of that event should be consecutive, without so much lag.

In order to prevent the contamination of blink and reduce the effect of random noise in F7 and F8 channels, F7 and F8 channels’ signals can be subtracted from each other. Since their behavior is opposite, this will increase the peak amplitudes while reducing the idle state amplitude, noise, and blink artifacts on those channels. This was actually tried with the offline case and it increased the accuracy of the offline detection results but since it was not tried for real-time application, it did not take place in this work. Moreover, Long Short Term Memory Recurrent Neural Networks, thanks to their memory units, showed good performances in different research that uses time domain signals for classification.

Future work will investigate transfer learning and meta-learning techniques (such as the approach in Ref.^46^) to generalize the training and improve the robustness of online performance. In addition, full-body motions (*e.g.*, the arms motion) will be also considered to improve the human-robot interaction naturalness and performance. Finally, software optimization will be investigated in order to minimize the online computational time, providing the user with a fast and natural human-robot interaction interface (Supplementary Video 1).

### Supplementary Information

Supplementary Video 1.

## Data Availability

The related code is available at the following link: https://github.com/KK4r4s/BCI-TIAGo-Control-with-EEG-Using-Eye-Artifacts. Data will be made available on reasonable request (write to loris.roveda@idsia.ch).
